# Cancer Immunoediting and beyond in 2021

**DOI:** 10.3390/ijms222413275

**Published:** 2021-12-10

**Authors:** Elena Monica Borroni, Fabio Grizzi

**Affiliations:** 1 Department of Medical Biotechnologies and Translational Medicine, University of Milan, 20133 Milan, Italy; elena.borroni@humanitasresearch.it; 2 Department of Immunology and Inflammation, IRCCS Humanitas Research Hospital, 20089 Rozzano, Italy

Human cancer has been depicted as a non-linear dynamic system that is discontinuous in space and time, but progresses through different sequential states (Figure 1) [1,2]. Cancer progression is coordinated by many processes and controls which operate over different scales ranging from molecular to environmental [2,3]. Moreover, each transformed cell acquires the capability to progress independently and non-linearly, i.e., it is a self-governing entity [4,5,6,7]. In contrast to linear systems, the behavior of non-linear systems may be recognized as unpredictable. Periods of inactivity may be punctuated by a sudden switch, i.e., apparent patterns may disappear and new patterns may suddenly emerge [2,8]. Non-linear systems do not react proportionally to the magnitude of their inputs depending on their initial conditions, i.e., small changes in the initial conditions may generate very different end-points, and their behavior is not deterministic [2]. It is known that human cancer does not conform to simple mathematical principles and that there are variable responses to therapeutic agents and divergent metastatic dynamics underlying very different clinical behaviors. Cancer outcomes also depend on the complex relations between tumor cells and other components in the tumor microenvironment, namely, tumor-infiltrating immune cells, fibroblasts, cancer stem cells, adipocytes, and endothelial cells [9,10]. As cancers advances, transformed cells can reshape the microenvironment to their advantage, often fueling inflammation and pro-tumorigenic microenvironmental crosstalk. In 1909, the immunologist Paul Ehrlich postulated that the immune system can identify and destroy early tumors in the absence of therapeutics. The term “cancer immunoediting” was introduced to define a dynamic process wherein immunity functions not only as an extrinsic tumor suppressor but also to shape tumor immunogenicity. It evolves through three sequential *states* that function either independently or in sequence and are called: (a) elimination, (b) equilibrium, and (c) escape (Figure 1) [11]. During the elimination phase, innate and adaptive immune systems cooperate with each other to detect and eliminate transformed cells before they become clinically evident. Malignant cells may, however, not be completely eradicated but instead enter into an equilibrium phase in which the immune system controls the tumor cell growth. In this phase, adaptive immunity constrains the growth of clinically undetectable tumor cells and edits tumor cell immunogenicity. Equilibrium is likely the longest of the three phases and may occur over a period of years. The tumor dormancy may, however, be suddenly interrupted, leading to progression of the cells into the escape phase, during which edited tumors of poor immunogenicity, begin to grow progressively in an immunologically unrestrained manner, establish an immunosuppressive tumor microenvironment, and lastly become clinically detectable (Figure 1). This breach of the host’s immune system most likely occurs when genetic and epigenetic changes in the tumor cell confer resistance to immune recognition and elimination, allowing the tumors to colonize distant sites.

Increasing evidence confirmed a basic role of chemokines and their cognate receptors in the migration pattern of immune cells into the tumor, thereby shaping the tumor microenvironment immune profile [12]. Chemokines act as key determinants of disease progression, with a strong influence on patient prognosis and response to therapy. Due to their multifaceted role in the tumor immune response, the chemokine network has emerged as a new potential immunotherapy target [13,14]. It is now also evident that cancer initiation and progression require a metabolic reprogramming of cancer cells. Transformed cells independently alter their flux through various metabolic pathways in order to meet the increased bioenergetic and biosynthetic demand as well as mitigate oxidative stress required for cancer cell proliferation and survival [15]. At the same time, a metabolic program is required to allow immune cells to acquire and utilize nutrients necessary for their survival, proliferation, and functions. The above considerations suggest that approaching human cancer and addressing its complexity in both time and space is likely to reveal more about its onset, progression and metastasis, and this manner of thinking may help to interpret experimental findings and categorize the rich body of knowledge on the basis of the similarities and/or shared behaviors of very different tumors. In this Special Issue titled “Cancer Immunoediting and beyond 2.0”, we aim at gathering a collection of original investigations and reviews into the different phases of “cancer immunoediting”, the complex tumor microenvironment and the cancer metabolism, covering a range of model systems and tumor types.

## Figures and Tables

**Figure 1:**
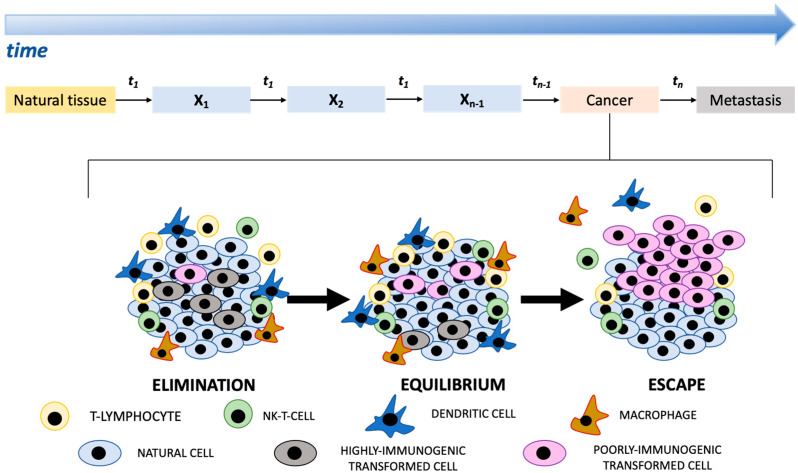
The cartoon shows the progression of different states (x_1_, x_2_, …, x_n−1_, x_n_) identifiable in the development of cancer from natural tissue. The exact time interval (t) between two successive states is unpredictable. Understanding how the immune system affects cancer progression still remains one of the most important topics in cancer immunology. Elimination, equilibrium, and escape represent three states that function either independently or in sequence in which a different relation between transformed cells and the immune system is established. The entire process is called “cancer immunoediting”.
